# European Validation of the Self-Evaluation of Negative Symptoms (SNS): A Large Multinational and Multicenter Study

**DOI:** 10.3389/fpsyt.2022.826465

**Published:** 2022-01-31

**Authors:** Sonia Dollfus, Armida Mucci, Giulia M. Giordano, István Bitter, Stephen F. Austin, Camille Delouche, Andreas Erfurth, W. Wolfgang Fleischhacker, Larisa Movina, Birte Glenthøj, Karoline Gütter, Alex Hofer, Jan Hubenak, Stefan Kaiser, Jan Libiger, Ingrid Melle, Mette Ø. Nielsen, Oleg Papsuev, Janusz K. Rybakowski, Gabriele Sachs, Alp Üçok, Francesco Brando, Pawel Wojciak, Silvana Galderisi

**Affiliations:** ^1^Service de Psychiatrie, CHU de Caen, Caen, France; ^2^UFR de Médecine, UNICAEN, Normandie Université, Caen, France; ^3^ISTS, UNICAEN, Normandie Université, Caen, France; ^4^Department of Psychiatry, University of Campania Luigi Vanvitelli, Naples, Italy; ^5^Department of Psychiatry and Psychotherapy, Semmelweis University, Budapest, Hungary; ^6^Psychiatric Research Unit, Region Zealand Psychiatry, Slagelse, Denmark; ^7^1st Department of Psychiatry and Psychotherapeutic Medicine, Klinik Hietzing, Vienna, Austria; ^8^Division of Psychiatry I, Department of Psychiatry, Psychotherapy, Psychosomatics and Medical Psychology, Medical University Innsbruck, Innsbruck, Austria; ^9^Department of Psychotic Spectrum Disorders, Moscow Research Institute of Psychiatry, Moscow, Russia; ^10^Center for Neuropsychiatric Schizophrenia Research (CNSR) and Center for Clinical Intervention and Neuropsychiatric Schizophrenia Research (CINS), Mental Health Center Glostrup, Glostrup, Denmark; ^11^Department of Clinical Medicine, Faculty of Health and Medical Sciences, University of Copenhagen, Copenhagen, Denmark; ^12^Department of Psychiatry and Psychotherapy, Psychiatric Hospital, University of Zurich, Zurich, Switzerland; ^13^Psychiatric Department, Charles University Medical School and Faculty Hospital Hradec Králové, Hradec Králové, Czechia; ^14^Adult Psychiatry Division, Department of Psychiatry, University of Geneva Hospitals, Geneva, Switzerland; ^15^NORMENT Centre, Institute of Clinical Psychiatry, Oslo University Hospital, University of Oslo, Oslo, Norway; ^16^Department of Adult Psychiatry, Poznan University of Medical Sciences, Poznan, Poland; ^17^Department of Psychiatry and Psychotherapy, Medical University of Vienna, Vienna, Austria; ^18^Psychotic Disorders Research Program, Istanbul Faculty of Medicine, Istanbul University, Istanbul, Turkey

**Keywords:** self-assessment, SNS, schizophrenia, negative symptoms, BNSS, confirmatory factor analysis

## Abstract

**Background:**

Negative symptoms are usually evaluated with scales based on observer ratings and up to now self-assessments have been overlooked. The aim of this paper was to validate the Self-evaluation of Negative Symptoms (SNS) in a large European sample coming from 12 countries. We wanted to demonstrate: (1) good convergent and divergent validities; (2) relationships between SNS scores and patients' functional outcome; (3) the capacity of the SNS compared to the Brief Negative Symptom Scale (BNSS) to detect negative symptoms; and (4) a five-domain construct in relation to the 5 consensus domains (social withdrawal, anhedonia, alogia, avolition, blunted affect) as the best latent structure of SNS.

**Methods:**

Two hundred forty-five subjects with a DSM-IV diagnosis of schizophrenia completed the SNS, the Positive and Negative Syndrome Scale (PANSS), the BNSS, the Calgary Depression Scale for Schizophrenia (CDSS), and the Personal and Social Performance (PSP) scale. Spearman's Rho correlations, confirmatory factor analysis investigating 4 models of the latent structure of SNS and stepwise multiple regression were performed.

**Results:**

Significant positive correlations were observed between the total score of the SNS and the total scores of the PANSS negative subscale (*r* = 0.37; *P* < 0.0001) and the BNSS (*r* = 0.43; *p* < 0.0001). SNS scores did not correlate with the level of insight, parkinsonism, or the total score of the PANSS positive subscale. A positive correlation was found between SNS and CDSS (*r* = 0.35; *p* < 0.0001). Among the 5 SNS subscores, only avolition subscores entered the regression equation explaining a lower functional outcome. The 1-factor and 2-factor models provided poor fit, while the 5-factor model and the hierarchical model provided the best fit, with a small advantage of the 5-factor model. The frequency of each negative dimension was systematically higher using the BNSS and the SNS vs. the PANSS and was higher for alogia and avolition using SNS vs. BNSS.

**Conclusion:**

In a large European multicentric sample, this study demonstrated that the SNS has: (1) good psychometric properties with good convergent and divergent validities; (2) a five-factor latent structure; (3) an association with patients' functional outcome; and (4) the capacity to identify subjects with negative symptoms that is close to the BNSS and superior to the PANSS negative subscale.

## Introduction

Negative symptoms are usually evaluated with scales based on observer ratings (as named hetero-assessment, HA) and up to now self-assessments (SA) have been overlooked, probably because of the idea that patients with schizophrenia with negative symptoms are unable to accurately report their own symptoms (1, 2). Indeed, only five self-report tools have been introduced (3); only two of them have been considered as self-assessments *sensu stricto*, the Motivation and Pleasure Scale–Self-Report (MAPSR) (4) and the Self-evaluation of Negative Symptoms (SNS) (5). In contrast, 18 HA scales (6) have been developed with the two most recent scales being the Brief Negative Symptom Scale (BNSS) (7) and the Clinical Assessment Interview for Negative Symptoms (CAINS) (8). Nevertheless, SA have several advantages over HA. They are easier to understand, more time-efficient since they need less time for the evaluation, they better assess subjective feelings and increase a patient's involvement in the treatment, and they might be more appropriate to detect the symptoms during a first psychotic episode (9) at the beginning or even before the onset of illness (10).

However, similar to HA scales, SA scales, using traditional or innovative methods (11), need to present good psychometric properties in order to be used them in therapeutic trials or in clinical practice. In the first and main validation study, the SNS demonstrated a good test-retest reliability, good internal consistency, and tight convergent and divergent validities (5). Other studies in French, American, and Polish populations confirmed similar good psychometric properties (12–14). A SNS total score threshold at 7 for the identification of the negative dimension of schizophrenia with good sensitivity and specificity has also been established (15). Arabic and Chinese versions of the SNS in patients with schizophrenia were also used showing that all items converged over a solution of five factors (16, 17). Despite all these studies were conducted in different countries (France, US, Spain, Poland, China, and Lebanon) further validation studies of the SNS are necessary, particularly on larger samples and in various European Countries. Therefore, the aim of this paper was to validate the SNS in a large European sample coming from 12 countries. First, we demonstrated good convergent and divergent validities. Of note, the relationships between the SNS and the BNSS, which is a valid and reliable instrument for assessing negative symptoms of schizophrenia across cultures (18), needed to be explored since most of the previous studies with the SNS tested convergent validity with the negative subscale of the Positive and Negative Syndrome Scale (PANSS) (16, 19) or with the Scale for the Assessment of Negative Symptoms (SANS) (5, 12, 20). Second, we tested the relationships between the SNS and patients' functional outcome and particularly the load of avolition since strong correlations between this dimension and functional outcome has previously been reported (21–24). Third, we evaluated the capacity of the SNS compared to the BNSS to detect negative symptoms by investigating the frequency of negative symptoms in a large sample. Fourth, we showed that a five-domain construct in relation to the 5 consensus domains (social withdrawal, anhedonia, alogia, avolition, blunted affect) was the best latent structure of the SNS since recent studies using the BNSS suggested that this model has better fit statistics than a 2-dimensional construct (25).

## Methods

### Participants

Study participants were recruited among subjects attending the outpatient and inpatient units of the Psychiatric Departments of 12 European centers (23) (see Appendix for details on the centers). Recruitment was carried out from October 31, 2016 to July 15, 2017. The instruction was to include around 20 patients by center. However, some centers could not reach this number during this short period of inclusion. Inclusion criteria were a diagnosis of schizophrenia according to DSM-IV, confirmed by the Mini Neuropsychiatric Interview-Plus (MINI-Plus); at the time of the study, the DSM-5 or the corresponding MINI was not available in all European countries. All the patients, in-patients included, were stable patients and their age was between 18 and 65 years. Exclusion criteria were: (a) treatment modifications and/or hospitalization due to symptom exacerbation in the last 3 months; (b) a history of moderate to severe intellectual disability or of neurological diseases; (c) a history of alcohol and/or substance abuse in the last 6 months; (d) current pregnancy; and (e) inability to provide informed consent. All participants signed written informed consent to participate in the study, after receiving detailed explanation of the study procedures and goals.

The local Ethics Committee of the involved institutions approved the study, and the study was performed in accordance with the ethical standards laid down in the 1964 Declaration of Helsinki.

### Assessment

The assessments used in the present study have been described previously (23). They included the BNSS (7), the PANSS (19), the Calgary Depression Scale for Schizophrenia (CDSS) (26), the St. Hans Rating Scale for Extrapyramidal Syndromes (SHSR) (27), and the Personal and Social Performance (PSP) scale (28). BNSS is a semi-structured interview with 13 items, composed of 5 negative symptom subscales (anhedonia, asociality, avolition, blunted affect and alogia), plus one item assessing the lack of the normal experience of distressing, unpleasant emotions. PANSS includes a subscale for positive symptoms with 7 items; a subscale for negative symptoms with 7 items, and a general psychopathology subscale with 16 items. CDSS is a 9-item scale evaluating depressive symptoms. SHSR was used to quantify the parkinsonism and PSP assessed real-life functioning with higher scores indicating better functioning. In addition to these HA evaluations, self-assessment of negative symptoms was carried out with the Self-evaluation of Negative Symptoms (SNS) (5).

The SNS, filled by the patients themselves, includes a form for patients with 20 items and a score sheet. Items are concise and easily understandable as reported by patients (14), and all items cover the five domains of negative symptoms, blunted affect being replaced by diminished emotional range (or emotional feeling). Most of the SNS items came from patient verbatim. All items are focused on internal experiences in order to be complementary with other scales based on observer ratings. To rate each item, the patient puts a cross in the box next to the response that best corresponds to his/her current feelings based on the previous week, scoring 2 (strongly agree), 1 (somewhat agree), or 0 (strongly disagree). The number of responses was voluntarily limited to three to simplify completion and avoid random responses when score ranges are too broad. The total score is the sum of the 20 items, ranging from 0 (no negative symptoms) to 40 (severe negative symptoms). The SNS is constituted of 5 subscales containing items investigating social withdrawal (items 1 to 4), reduced emotional range (items 5 to 8), alogia (items 9 to 12), avolition (items 13 to 16), and anhedonia (items 17 to 20). Patients generally complete the questionnaire in <5 min. The current version of the SNS evaluates the five sub-domains as follows: *Social withdrawal* assesses social, family, and friend relationships as well as the patient's desire to establish new relationships; *Diminished emotional range* evaluates happiness or sadness as perceived by the patient in situations in which happiness or sadness is usually felt; *Avolition* is assessed by the patient's difficulty with the goals he/she has set with respect to consistency in activities of daily life, his/her desire, his/her motivation, and energy; *Anhedonia* evaluates the pleasure perceived by the patient with those around him/her, consummatory and anticipatory pleasure; *Alogia* is assessed by patient's perception and the efforts required by the patient to interact.

All non-French versions of the SNS were developed using the translation–back translation method. The translated versions were back-translated into French by a native speaker of the same language in which the scale was used. The back-translated versions were reviewed and approved by the author who created the scale (5).

### Data Analyses

#### Convergent and Divergent Validity of SNS

Correlation analyses were performed to examine the convergent validity of the SNS with other scales assessing negative symptoms (i.e., BNSS and PANSS negative subscale) and to examine the discriminant validity of the SNS with measures of insight as well as depressive, positive, and extrapyramidal symptoms (i.e., Lack of judgement and insight out of the PANSS (G12), CDSS, PANSS positive subscale, and parkinsonism scores of the SHRS, respectively).

Spearman's Rho correlations were used. As with large sample size (*N* > 200), *p-*values are generally highly significant even for very low correlation coefficients (*r* = 0.10, *p* < 0.01), the absolute value of the correlation coefficient is a more meaningful estimation of the association. Correlation coefficients (in absolute value) <0.35 are generally considered to represent low or weak correlations, those from 0.36 to 0.67 modest to moderate correlations, and those from 0.68 to 1.0 strong correlations (29).

#### Confirmatory Factor Analyses of SNS

**Figure 1:**
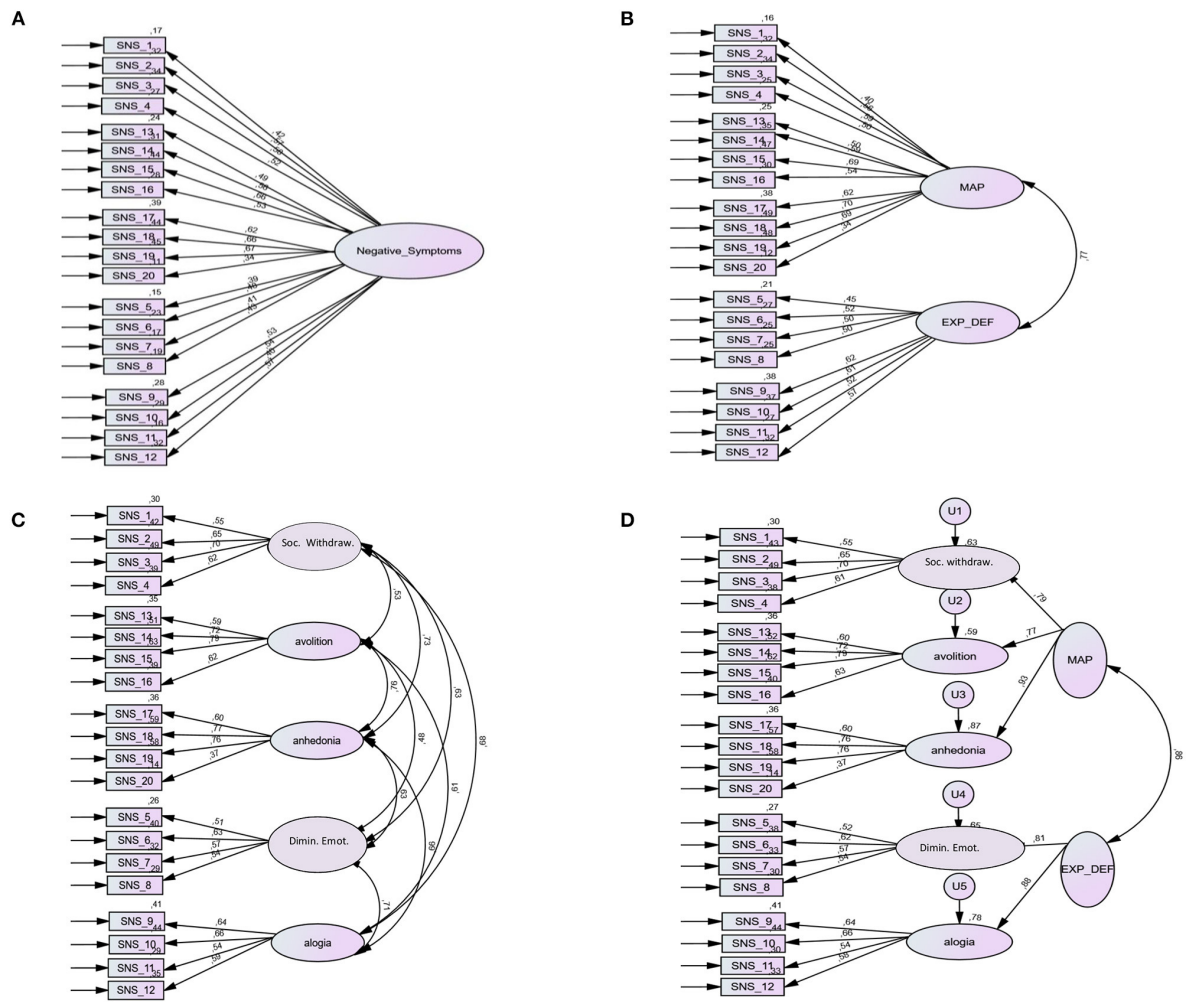
One-factor, two-factor, five-factor, and hierarchical models of the Self-evaluation of Negative Symptoms (SNS). **(A)** The one-factor model encompasses all 20 items of SNS. **(B)** The two-factor model corresponds to the motivation and pleasure (MAP) and expressive deficit (EXP_DEF) factors (MAP includes the 12 items coming from anhedonia, avolition, and social withdrawal subscales of SNS; EXP_DEF includes the eight items coming from alogia and diminished emotional range subscales of SNS). **(C)** The five-factor model corresponds to the five subscales of SNS: social withdrawal (items 1–4), diminished emotional range (items 5–8), alogia (items 9–12), avolition (items 13–16), and anhedonia (items 17–20) subscales. **(D)** The hierarchical model consists of the five factors and the second-order factors of motivation and pleasure (MAP) and expressive deficit (EXP_DEF). Solid lines represent factor loadings and curved lines represent the correlation among factors. Numbers indicate item numbers of the SNS. Soc. Withdraw., social withdrawal; Dimin. Emot., diminished emotional range.

Confirmatory factor analysis (CFA) was used to test 4 models of the latent structure of negative symptoms (Figure 1) according to previous data from HA scales for negative symptoms (23, 25, 30). The tested models included: one factor—encompassing all 20 items of the SNS; two-factor model corresponding to the expressive deficit (EXP_DEF) and the motivation and pleasure (MAP) factors (EXP_DEF including the 8 items coming from the alogia and the reduced emotional range subscales of the SNS; MAP including the 12 items coming from the anhedonia, the avolition, and the social withdrawal subscales of the SNS); a five-factor model corresponding to the 5 subscales of the SNS; a hierarchical model with 2 second-order factors corresponding to EXP_DEF and MAP as well as the 5 first-order factors reflecting the 5 aforementioned subscales.

To assess the absolute fit of the models, the following indices were used: χ^2^ value, the comparative fit index (CFI), the Tucker Lewis index (TLI), and the root mean square error of approximation (RMSEA). A good fit included a non-significant χ^2^ value, CFI and TLI values ≥0.95, and RMSEA ≤ 0.08. Two information criteria, the Akaike information criterion (AIC) and the Browne-Cudeck criterion (BCC), were used to compare relative fit indices of model parsimony. Lower values indicate better model fit.

#### Association of SNS With Functional Outcome

The association of negative symptoms with patients' functional outcome was studied using multiple stepwise linear regression. Stepwise multiple regressions were conducted with the PSP total score as the dependent variables and the 5 SNS subscores, the PANSS positive subscores, disorganization and insight item scores, male gender, duration of illness, age, educational level, and the CDSS scores as independent variables; the choice of these predictors was based on our previous results (23). To deal with potential multicollinearity, the variance inflation index (VIF) was determined for each variable included in the model, a value above 5 indicating critical collinearity.

#### Assessment of Frequency of at Least Moderate Severity Negative Symptoms

As the European Medicines Agency (EMA) guidelines on clinical trial design for negative symptoms require the inclusion of subjects with at least moderate severity of negative symptoms, defined on an accepted and validated rating scale, we investigated the frequency of these symptoms using the SNS, the BNSS, and the PANSS negative subscale. For the 5 SNS subscales, a score ≥2 was required on the subscores, whereas for the 5 BNSS subscales, a score ≥3 (moderate) was necessary on the subscores. For the PANSS, a score ≥4 (moderate) on any of the following core negative symptoms was taken into account: Blunted Affect (N1), Emotional Withdrawal (N2), Poor Rapport (N3), Passive Social Withdrawal (N4), and Lack of Spontaneity (N6).

Type I error was 5%. All analyses were performed on IBM SPSS 22.0 software except the CFA (maximum likelihood) that was estimated using AMOS 21.0.

## Results

Two hundred forty-five subjects with a DSM-IV diagnosis of schizophrenia completed the SNS. Data on demographic and illness related variables in the whole sample and for the 12 centers are provided in Tables 1, 2.

**Table 1:** Demographic characteristics in all the sample (*N* = 245) and in each center.

	**C01**	**C02**	**C04**	**C05**	**C06**	**C10**	**C11**	**C12**	**C14**	**C15**	**C16**	**C17**	**All sample**
	**(*N* = 25)**	**(*N* = 20)**	**(*N* = 20)**	**(*N* = 24)**	**(*N* = 32)**	**(*N* = 25)**	**(*N* = 7)**	**(*N* = 19)**	**(*N* = 24)**	**(*N* = 20)**	**(*N* = 20)**	**(*N* = 9)**	**(*N* = 245)**
*N* (%)													
Gender (male)	14 (56)	10 (50)	14 (70)	17 (70.8)	26 (81.3)	13 (52)	2 (28.6)	9 (47.4)	18 (75)	11 (55)	14 (70)	7 (77.8)	155 (63)
Antipsychotic treatment	25 (100)	20 (100)	20 (100)	14/20 ^*^(70)	31 (97)	24 (96)	7 (100)	19 (100)	24 (100)	18 (90)	20 (100)	9 (100)	241 (95.8)
Mean (SD)													
Age (years)	44.68 (11.58)	34.95 (12.89)	39.60 (10.35)	29.67 (8.44)	38.69 (8.88)	43.32 (8.92)	38.71 (5.85)	40.42 (11.72)	31.71 (9.96)	35.35 (10.27)	37.15 (11.45)	32.11 (16.57)	37.4 (11.3)
Education (completed years)	13.52 (3.94)	14.37 (4.39)	12.15 (2.47)	12.37 (3.46)	12.62 (2.33)	12.72 (3.07)	12.00 (3.05)	13.00 (2.76)	12.14 (3.62)	14.95 (2.62)	12.35 (2.27)	11.44 (2.60)	12.9 (3.2)
Duration of illness (years)	14.16 (9.46)	12.49 (9.12)	15.30 (11.11)	6.42 (4.40)	13.53 (8.84)	18.68 (7.48)	10.71 (0.75)	13.10 (8.54)	7.81 (7.82)	13.85 (8.98)	14.00 (9.89)	5.16 (8.97)	12.6 (9.1)

* *4 missing data*.

**Table 2:** Clinical variables in all the sample (N = 245) and in each center [mean (SD)].

	**C01**	**C02**	**C04**	**C05**	**C06**	**C10**	**C11**	**C12**	**C14**	**C15**	**C16**	**C17**	**All sample**
	**(*N* = 25)**	**(*N* = 20)**	**(*N* = 20)**	**(*N* = 24)**	**(*N* = 32)**	**(*N* = 25)**	**(*N* = 7)**	**(*N* = 19)**	**(*N* = 24)**	**(*N* = 20)**	**(*N* = 20)**	**(*N* = 9)**	**(*N* = 245)**
PANSS total	55.36 (15.98)	64.75 (14.47)	74.80 (14.29)	62.42 (17.13)	72.81 (13.32)	64.92 (20.07)	56.43 (21.73)	52.95 (12.44)	47.33 (12.08)	61.35 (14.23)	53.95 (12.40)	55.33 (14.75)	61.1 (16.9)
PANSS positive	11.76 (4.74)	14.55 (5.97)	16.20 (4.84)	15.17 (5.76)	14.66 (5.43)	14.04 (5.95)	13.14 (5.17)	11.79 (3.22)	8.50 (1.56)	12.30 (3.26)	11.50 (5.19)	12.67 (5.14)	13.1 (5.2)
PANSS negative	15.48 (6.76)	16.90 (5.06)	22.30 (5.44)	17.25 (4.96)	20.09 (5.46)	19.80 (9.38)	14.71 (9.05)	15.21 (6.67)	15.75 (7.15)	17.60 (4.81)	14.85 (4.14)	14.33 (5.45)	17.4 (6.6)
PANSS disorganization	1.92 (1.22)	2.40 (1.35)	2.95 (1.19)	2.25 (1.32)	1.78 (1.03)	2.32 (1.72)	1.43 (0.78)	1.72 (0.66)	1.42 (0.83)	2.20 (0.83)	1.80 (0.83)	1.89 (1.05)	2.03 (4.2)
PANSS abstract thinking item	2.20 (1.35)	2.85 (1.53)	2.95 (1.35)	3.25 (1.32)	2.66 (1.18)	3.16 (2.13)	1.67 (1.2)	2.05 (1.39)	1.71 (1.04)	2.65 (0.81)	1.85 (1.13)	2.67 (1.22)	2.53 (1.4)
PANSS Insight item	1.76 (1.16)	2.05 (1.19)	3.45 (1.35)	2.13 (1.36)	3 (1.04)	2.28 (1.37)	2.00 (1.15)	1.37 (0.59)	1.08 (0.48)	2.40 (0.94)	2.42 (1.12)	1.89 (1.05)	2.19 (1.2)
BNSS total	15.20 (11.51)	23.60 (11.11)	33.10 (12.80)	17.83 (8.17)	33.47 (13.55)	34.28 (19.05)	25.29 (18.68)	21.58 (17.00)	26.63 (17.86)	24.75 (12.92)	34.80 (13.34)	16.67 (8.12)	26.3 (15.4)
SNS total	10.80 (5.76)	19.50 (8.81)	17.90 (6.68)	15.29 (6.58)	16.22 (9.37)	11.80 (8.82)	16.43 (9.27)	16.74 (11.04)	14.38 (6.89)	10.70 (6.27)	15.65 (6.61)	13.22 (6.45)	14.8 (8.1)
Withdrawal subscale	1.96 (1.56)	3.85 (2.32)	2.90 (1.80)	3.29 (2.13)	2.63 (2.42)	1.96 (2.50)	3.71 (2.49)	3.26 (2.44)	2.63 (1.58)	2.05 (1.73)	3.00 (2.12)	2.22 (1.92)	2.7 (2.1)
Diminished emotional range subscale	2.88 (1.96)	3.30 (2.22)	3.95 (1.79)	2.75 (2.02)	2.84 (2.05)	2.76 (2.26)	3.71 (2.21)	3.32 (2.38)	3.25 (1.93)	1.60 (1.18)	4.25 (2.38)	3.00 (2.39)	3.1 (2.1)
Alogia subscale	2.32 (2.15)	4.50 (2.66)	4.35 (1.78)	2.88 (1.70)	3.94 (2.34)	3.08 (2.15)	2.86 (2.34)	3.68 (2.54)	2.96 (2.17)	2.25 (1.71)	3.10 (1.83)	3.11 (2.47)	3.2 (2.2)
Avolition subscale	2.08 (1.73)	4.05 (1.87)	3.90 (2.24)	3.38 (2.61)	4.31 (2.49)	2.36 (2.23)	3.29 (1.79)	3.74 (2.78)	3.25 (1.87)	2.70 (2.20)	2.70 (1.80)	2.67 (2.44)	3.2 (2.2)
Anhedonia subscale	1.56 (1.50)	3.80 (2.16)	2.80 (2.14)	3.00 (1.91)	2.50 (2.51)	1.64 (1.72)	2.86 (2.11)	2.74 (2.64)	2.29 (1.75)	2.10 (1.68)	2.60 (1.84)	2.22 (1.39)	2.5 (2)
CDSS total	3.16 (3.78)	6.70 (4.10)	2.55 (3.73)	2.75 (3.32)	4.78 (3.59)	4.32 (3.32)	4.29 (5.73)	3.68 (3.25)	2.08 (2.73)	2.80 (2.39)	1.65 (1.59)	2.89 (2.42)	3.5 (3.5)
SHRS	5.52 (4.87)	2.50 (3.44)	3.70 (4.11)	1.67 (2.07)	10.16 (5.72)	2.12 (5.01)	3.00 (5.13)	0.79 (1.08)	0.29 (0.90)	3.45 (3.56)	6.35 (5.71)	2.44 (2.35)	3.8 (5.0)
PSP total	58.92 (14.13)	55.30 (15.05)	45.25 (13.04)	59.54 (11.05)	53.56 (18.29)	61.84 (9.82)	NA	65.53 (21.54)	53.79 (16.44)	61.25 (12.53)	66.00 (7.93)	54.33 (8.54)	57.4 (15.3)

*CDSS, calgary depression scale for schizophrenia; BNSS, brief negative symptom scale; PANSS, positive and negative syndrome scale; PSP, personal and social performance; SHSR, St. Hans rating scale for extrapyramidal syndromes, parkinsonism total; SCZ, subjects with schizophrenia; SNS, Self-evaluation of negative symptoms; NA, not available*.

### Convergent and Divergent Validity

#### Analyses in the Whole Sample

Significant positive correlations were observed between the total scores of the SNS and the total scores of negative PANSS subscale (*r* = 0.37; *P* < 0.0001) and BNSS (*r* = 0.43; *p* < 0.0001) although the coefficients were considered as moderate (Table 3).

**Table 3:** SNS convergent and discriminant validity (rho-values).

	**SNS total score**	***p-*value**
**Convergent validity**		
PANSS negative subscore	0.37	<0.0001
BNSS total score	0.43	<0.0001
**Discriminant validity**		
PANSS positive subscore	0.12	NS
CDSS total score	0.35	<0.0001
Lack of judgement and insight (PANSS item)	−0.02	NS
Parkinsonism (SHSR)	0.05	NS

*NS, no significant*.

SNS scores did not correlate with the level of insight (*r* = −0.02), Parkinsonism (*r* = 0.05), or with the positive PANSS scores (*r* = 0.12). A positive correlation was found between SNS and CDSS (*r* = 0.35; *p* < 0.0001). A stepwise multiple regression with CDSS as dependent variable and the 5 subscores of SNS as independent variables showed that the model was significant (adjusted *R*^2^ = 0.179; *p* < 0.0001) and accounted for 17.9% of the variance of the CDSS total score. Higher depression was associated with higher avolition scores which accounted for most of the explained CDSS variance (14.3/17.9%). Moreover, among the 5 subscores of SNS, the reduced emotional range subscores compared to other subscores of SNS presented the lowest correlation with CDSS scores (*r* = 0.14, *p* = 0.02).

#### Analyses by Center

Ten out of 12 centers displayed correlations between SNS and BNSS scores (from *r* = 0.42, *p* = 0.06 to *r* = 0.72, *p* < 10^−3^) (Table 4). Patients included in the two remaining centers (C04 and C010) displayed correlations <0.3 between the SNS and the BNSS.

**Table 4:** Correlations between SNS and other scales in each center.

**Centers**	***N***	**BNSS**	**PANSS negative**	**PANSS positive**	**CDSS**	**Insight^**a**^**	**SHRS^**b**^**
C01	25	0.64^***^	0.41^*^	0.22	0.07	−0.07	0.27
C02	20	0.54^**^	0.56^*^	−0.09	0.39	0.16	−0.19
C04	20	0.29	0.19	0.02	0.48^*^	−0.14	−0.2
C05	24	0.41^*^	0.40^*^	0.21	0.33	0.14	0.06
C06	32	0.45^**^	0.39^*^	−0.08	0.33	0.07	0.06
C10	25	0.12	0.04	−0.19	0.25	−0.21	0.17
C11	7	0.75^*^	0.58	0.07	0.74 (*p* = 0.057)	−0.02	−0.03
C12	19	0.62^***^	0.32	0.51^*^	0.76^****^	−0.49^*^	0.28
C14	24	0.72^****^	0.68^****^	0.34	0.43^**^	−0.35 (*p* = 0.09)	0.14
C15	20	0.42 (*p* = 0.06)	0.34	0.61^**^	0.47^*^	−0.25	−0.10
C16	20	0.47^*^	0.29	−0.14	0.47^*^	−0.19	0.33
C17	9	0.47	0.41	0.05	0.19	0.17	−0.06
Total	245						

* *p < 0.05;*

** *p = 0.01;*

*** *p = 0.001;*

**** *p < 0.0001*.

a *G12 PANSS item*.

b *Parkinsonism score*.

The correlations between the SNS and the PANSS negative subscale were weaker than those between the SNS and the BNSS and were observed in 7 centers (from *r* = 0.41 to *r* = 0.68). No center achieved correlations between SNS and SHRS scores. Only one center reported a significant correlation between the SNS and the PANSS positive subscale (*r* = 0.51, *p* < 0.05). Seven centers reported correlations between SNS and CDSS scores (from *r* = 0.39, NS to *r* = 0.76, *p* < 10^−3^). All centers except one reported the absence of any correlation between SNS and the insight PANSS item.

### Confirmatory Factor Analyses (CFA)

The four models are displayed on Figure 1. Results of the CFA analyses are reported in Table 5. The 1-factor and 2-factor models provided poor fit, while the 5- factor model and the hierarchical model provided the best fit, with a small advantage of the 5-factor model. In summary, both the 5-factor and the hierarchical models are supported by our study.

**Table 5:** Model fit results from CFA on negative symptoms as assessed by the SNS.

**Model**	**Number of distinct parameters to be estimated**	**AIC**	**BCC**	**X^2^ Value (df)**	**TLI**	**CFI**	**RMSEA**
1 factor	60	580,259	591,559	460,259 (170)	0.733	0.783	0.084
2 factor	61	523,157	534,646	401,157 (169)	0.785	0.827	0.075
5 factor	70	390,202	403,385	250,202 (160)	0.912	0.933	0.048
Hierarchical	66	392,638	405,069	260,638 (164)	0.908	0.928	0.049

*CFA, confirmatory factor analysis; AIC, akaike information criterion; BIC, browne-cudeck criterion; CFI, confirmatory fit index; RMSEA, root mean square error of approximation; TLI, tucker lewis index*.

### Association of SNS With Functional Outcome

Stepwise multiple regression revealed that 4 variables significantly explained the PSP scores: the PANSS positive subscale, the SNS avolition subscale, the educational level, and the lack of judgement and insight PANSS items (Table 6). The model was significant (*R*^2^ = 0.234, *p* < 0.0001) explaining 23.4% of the variance of PSP scores. All the VIF values were largely below the threshold of 5 (from 1.07 for gender to 2.63 for PANSS positive sub-scores), indicating no multicollinearity among independent variables. Among the 5 SNS subscores, only avolition subscores entered the regression equation. Lower functional outcome was predicted by higher positive dimension scores, avolition scores, lower levels of school education, and higher levels of lack of judgement and insight (β = −0.263, *p* < 0.0001, β = −0.218, *p* < 0.0001, β = 0.162, *p* =0.006, β = −0.152, *p* = 0.025, respectively). The other independent variables (age, duration of illness, disorganization PANSS item, the other negative dimensions, and depression) did not enter in the regression equation.

**Table 6:** Results of stepwise multiple regression on Personal and Social Performances (PSP) scores (dependent variable) in patients with schizophrenia.

	**Model**	**Beta**	***p***	**Adjusted *R* square**
1	PANSS positive-P1-P7	−0.393	<0.0001	0.151^a^
2	PANSS positive-P1-P7	−0.360	<0.0001	0.195^b^
	SNS-Avolition-items 13-16	−0.221	<0.0001	
3	PANSS positive-P1-P7	−0.339	<0.0001	0.220^c^
	SNS-Avolition-items 13-16	−0.209	0.001	
	School level (years)	0.170	0.005	
4	PANSS positive-P1-P7	−0.263	<0.0001	0.234^d^
	SNS-Avolition-items 13-16	−0.218	<0.0001	
	School level (years)	0.162	0.006	
	Lack of judgement and insight	−0.152	0.025	

a *Independent variable: PANSS positive subscores*.

b *Independent variables: PANSS positive subscores, SNS avolition subscores*.

c *Independent variables: PANSS positive subscores, SNS avolition subscores, school level*.

d *Independent variables: PANSS positive subscores, SNS avolition subscores, school level, lack of judgement and insight (PANSS G12 item)*.

### Frequency of at Least Moderate Severity Negative Symptoms

Frequency of at least moderate severity negative symptoms out of the five BNSS subscales, the 5 PANSS negative items, and the 5 SNS subscales in the whole sample are presented in Table 7. Two hundred and thirty-four study participants (95.5%) had a score ≥2 in at least one negative dimension from the 5 SNS subscales, while 227 (92.6%) had a score ≥3 in at least one negative dimension from the 5 BNSS subscales, and only 125 (51%) had a score ≥ 4 in at least one of the 5 PANSS items. The frequency of each negative dimension was systematically higher using the BNSS vs. the PANSS and was higher for alogia and avolition using the SNS vs. the BNSS.

**Table 7:** Frequency of negative symptoms of moderate severity in the whole study sample (*N* = 245).

**Negative symptoms (subscores)**	**SNS**		**BNSS**		**PANSS**		
	***N***	**(%)**	***N***	**(%)**	**Negative symptoms**	***N***	**(%)**
Social withdrawal	161	(65.7)	180	(73.4)	Passive/apathetic social withdrawal (N4)	61	(24.8)
Reduced emotion range	181	(73.8)	191	(77.9)	Blunted affect (N1)	95	(38.7)
Alogia	188	(76.7)	124	(50.6)	Lack of spontaneity (N6)	51	(20.8)
Avolition	177	(72.2)	171	(69.7)	Emotional withdrawal (N2)	60	(24.4)
Anhedonia	155	(63.2)	176	(71.8)	N/A		
N/A					Poor rapport (N3)	30	(12.2)
At least 1	234	(95.5)	227	(92.6)	At least 1	125	(51)
At least 2	213	(86.9)	205	(83.6)	At least 2	76	(31)
At least 3	185	(75.5)	174	(71)	At least 3	51	(20.8)

*BNSS, brief negative symptom scale; PANSS, positive and negative syndrome scale; SNS, self-evaluation of negative symptoms; N/A, not applicable*.

## Discussion

In a large European multicentric sample this study demonstrated that the SNS has: (1) good psychometric properties with good convergent and divergent validities; (2) a five-factor latent structure; (3) significant predictive power for functional outcome; and (4) the capacity to identify subjects with negative symptoms, close to the BNSS but superior to the PANSSnegative.

### Convergent and Divergent Validity

The results of the present study confirm those reported in a first validation study of the SNS (5). As previously observed with the Brief Psychiatric Rating Scale (BPRS) negative cluster (31), the correlation between the SNS and the PANSS negative subscale was modest but higher with the BNSS. In the present study, we demonstrated for the first time a significant correlation between the SNS and the BNSS that reinforces the good convergent valididity of the SNS already observed with the PANSS negative subscale, the BPRS negative cluster, or the SANS, scales that have been strongly criticized for no longer responding to the current conception of negative symptoms (32). We also observed no relationship between the SNS and positive symptoms, insight, and parkinsonism which also supports good discriminant validity since similar results were previously observed with different scales and samples. We also replicated the positive correlation between the SNS and depression assessed with the CDSS and demonstrated that the SNS avolition subscale mainly contributes to this correlation. At the same time, we have to point out that the SNS reduced emotional range subscale presented the lowest correlation with the CDSS which might suggest that reduced emotional feeling might be the best negative symptom for discriminating depressive symptoms as previously suggested (5). According to Delay (33), the mood in schizophrenia is reduced but exacerbated tinged with guilt and moral pain in depression and so hypothymia and hyperthymia, as called by Delay, characterizes schizophrenia, and depression, respectively. The absence of any correlation between the diminished emotional range subscale of the SNS and the blunted affect domain of the BNSS suggests a disjuntion between emotional expressiveness and emotional experience self-report. This is consistent with previous data (34) and with Bleuler's view on an inhibition model of affective flattening suggesting that patients with schizophrenia do experience emotion but the outward expression of these feelings is hidden (35). These findings might also contribute to the ongoing debate on the psychopathological construct of detachment within the hierarchical model of psychopathology (36). In fact, detachment is thought to be at the basis of emotional withdrawal as well as avolition, while our data suggest a disjunction. Whether the deficits in the outward expression of emotion are related to social cognitive impairment is not clear and deserves further investigation (37).

These results in the whole sample were also observed in the most centers. Indeed, 10 out of 12 centers found correlations between the SNS and the BNSS, while the correlations between the SNS and the PANSS negative subscale were weaker and were observed in only seven centers. No center reported correlations between the SNS and parkinsonism, and only one center reported correlations between the SNS and insight, while seven centers reported correlations between the SNS and depression. It is worth noting that patients from the different centers presented several different characteristics. For instance, patients from the centers C04 and C10 were older (except for one center), had the longest duration of illness, the highest scores on the PANSS, the PANSS negative subscale, the PANSS disorganization item, and the PANSS insight item (Table 2). Patient from the center C04 might correspond to severely ill schizophrenia patients with potentially secondary negative symptoms since these individuals had the highest scores on the PANSS positive subscale and the lowest scores on the PSP. Patient from the center C10 presented the highest scores on the PANSS abstract thinking item, which might have had an influence on self-assessment. This aspect should be confirmed in a further study investigating the cognitive functions in the same sample. In the same way, some discrepancies between the SNS and clinician-rated negative symptoms have previously been reported in patients with low levels of insights, with cognitive impairments, high antipsychotic dosages, and low functioning (14).

### Confirmatory Factor Analyses (CFA)

The confirmatory factor analysis of the SNS showed that the best latent structure was a five-domain construct in relation to the five consensus domains (social withdrawal, anhedonia, alogia, avolition, blunted affect) that exactly corresponds to the five sub-scales of the SNS with the correspondence of reduced emotional range to blunted affect. This result reinforces the questioning of the 2-dimensional construct and the idea that negative symptoms can be characterized by 5 rather than 2 dimensions (30). They also confirm recent findings with the BNSS suggesting that a five-domain construct might have the best fit statistics (23, 25). However, some previous results reported a 2-factor structure for negative symptoms with different scales, such as the Schedule for the Deficit Syndrome (SDS) (38, 39), the BNSS (7, 40–43), the CAINS (8) and the SNS (5). However, the present study explored the best fit of several structure models (one, two, 5-factor structure, and a hierachical model) using CFA, while in the previous study on SNS (5), an exploratory factor analysis was carried out on the 5 subscale scores of the SNS (and not on the 20 items) and without testing different structure models. The results of the present study confirm those reported elsewhere in different populations from different countries, in clinically stable outpatients with schizophrenia from Hong Kong (17), in hospitalized patients with schizophrenia from Lebanon (16), or in adolescents from the general population (10).

### Association of the SNS With Patients' Functional Outcome

Among the 5 SNS subscales, only the avolition subscale entered the regression model, explaining together with the positive subscores, the level of education, and the lack of judgement and insight PANSS item, 23.4% of the variance of functional outcome. This result again confirms that avolition is a major determinant of the functional outcome in agreement with several studies (22, 38, 44–46) and supports that SNS avolition subscale is related with real-life functioning.

### Frequency of at Least Moderate Severity Negative Symptoms

Our results also demonstrate the superiority of the SNS over the PANSS negative subscale to detect patients with at least one moderate-severity negative dimension (95.5 vs. 51%); this capacity is close to the BNSS (92.6%) but better for alogia and avolition, also with respect to the latter scale. However, comparing negative dimensions (corresponding to the subscales) including 2–4 items (for SNS and BNSS) with only one symptom (for the PANSS) weakens the results. In the same vein, the fact that the frequencies of alogia and avolition were higher with SNS than with BNSS may be due to the fact that the corresponding SNS subscales contain four items while the BNSS subscales only have two items.

Nevertheless, using SNS is promising in the detection of negative symptoms at the beginning of illness and even in patients with ultra high risk of psychosis as already suggested by several reports (9, 10). As negative symptoms appear early with a high prevalence compared to attenuated positive symptoms, it might be useful to detect them with SA. Identifying negative symptoms as soon as possible might lead fast therapeutic management and so might improve the functioning.

This paper presents some minor limitations. The number of patients included is small for some countries which did not allow us to test the validation of the SNS scale specifically in each country. The different patient groups were heterogeneous, notably for two countries where the patient characteristics differed, explaining probably the variability of some results. The inter-rater reliability across-centers was not assessed for this study. However, this concerns scales based on HA but not SA. The detection of negative symptoms with different scales and their comparisons need to be interpreted with caution since the number of items constituting the scales and subscales differ between each other.

These results as a whole support the European recommendations on the assessment of negative symptoms according to which SA can be used to complement HA (31). The SNS offers several advantages. It assesses the five negative dimensions required, is very simple and is easy to fill in by the patient since questions are very simple, with short sentences and easy choice in the answers. It requires patients' participation and improves their involvement in the treatment. Moreover, this scale is time-efficient, taking <5 min, much less than clinician's evaluations. This self-evaluation is complementary to the evaluation based on observer ratings; it provides clinical information not necessarily detected by caregivers or medical staff in a standard interview; in particular, it allows the patients to express their own feelings and symptoms as well as the level of awareness of symptoms. Thus, patients are not influenced by the questions of the rater and accordingly, their subjectivity is only concerned.

In conclusion, this European study demonstrates that SNS has good psychometric properties, a five-factor latent structure, the capacity to identify subjects with negative symptoms and that SNS is associated with patients' functional outcome.

## Data Availability Statement

The raw data supporting the conclusions of this article will be made available by the authors, without undue reservation.

## Ethics Statement

The studies involving human participants were reviewed and approved by Ethical Committee from the 12 countries. The patients/participants provided their written informed consent to participate in this study.

## Author Contributions

All authors listed have made a substantial, direct, and intellectual contribution to the work and approved it for publication.

## Funding

The European College of Neuropsychopharmacology (ECNP) supported the network standalone meetings during which the protocol was designed and results discussed by the involved researchers.

## Conflict of Interest

SD has been an expert and consultant or participated in educational conferences for the following industrial laboratories or companies: Gedeon Richter, Lundbeck Otsuka, Roche, Takeda, Fabre, Janssen, ONO Pharma, and Verasci. She also had a license agreement on SNS with MedAvante-ProPhase. AE received consulting fees and/or honoraria for speeches within the last 3 years from Angelini, AOP Orphan, Germania, Janssen, Krka, Lundbeck, Mylan, Neuraxpharm, Recordati, and Sandoz. SG has been a consultant and/or advisor to or has received honoraria from Millennium Pharmaceutical, Innova Pharma–Recordati Group, Janssen Pharmaceutica NV, Gedeon Richter-Recordati, Angelini, Lundbeck Italia, and Sunovion Pharmarmaceuticals. She has no other conflicts to disclose. AH has been a consultant for Recordati and participated in educational conferences for Janssen and Lundbeck. JH has received honoraria from Angelini, Lundbeck, and Servier. SK has received royalties for cognitive test and training software from Schuhfried. AM has been a consultant and/or advisor to or has received honoraria from Gedeon Richter Bulgaria, Janssen Pharmaceuticals, Lundbeck, Otsuka, Pfizer, and Pierre Fabre. GS is president of the Austrian Society of Neuropsychopharmacology and Biological Psychiatry, which is partially financed by the support from pharmaceutical companies. GS received consulting fees and/or honoraria for speeches within the last 3 years from Janssen, Lundbeck, Mylan, Recordati, and Schwabe. AÜ has been a consultant and/or advisor to or has received honoraria from Adeka, Janssen Pharmaceuticals, Abdi Ibrahim Otsuka, Sanovel, and Biofarma. The remaining authors declare that the research was conducted in the absence of any commercial or financial relationships that could be construed as a potential conflict of interest.

## Publisher's Note

All claims expressed in this article are solely those of the authors and do not necessarily represent those of their affiliated organizations, or those of the publisher, the editors and the reviewers. Any product that may be evaluated in this article, or claim that may be made by its manufacturer, is not guaranteed or endorsed by the publisher.

## Appendix

### List of the Centers Included in the Study

- Medical University Innsbruck, Department of Psychiatry, Psychotherapy Psychosomatics and Medical Psychology, Division of Psychiatry I, Innsbruck, Austria Department of Psychiatry and Psychotherapy, Medical University of Vienna, Vienna, Austria
- 1st Department of Psychiatry and Psychotherapeutic Medicine, Klinik Hietzing, Vienna, Austria, formerly 6th Psychiatric Department, Otto-Wagner-Spital, Vienna, Austria
- Psychiatric Department, Charles University Medical School and Faculty Hospital Hradec Králové, Hradec Králové, Czech Republic
- Center for Neuropsychiatric Schizophrenia Research (CNSR) and Center for Clinical Intervention and Neuropsychiatric Schizophrenia Research (CINS), Mental Health Center Glostrup, Glostrup, Denmark; University of Copenhagen, Faculty of Health and Medical Sciences, Dept. of Clinical Medicine, Copenhagen, Denmark
- Service de Psychiatrie, CHU de Caen, Caen, France
- Department of Psychiatry, University of Campania “Luigi Vanvitelli”, Naples, Italy
- NORMENT Centre, Institute of Clinical Psychiatry, University of Oslo and Oslo University Hospital, Oslo, Norway
- Department of Adult Psychiatry, Poznan University of Medical Sciences, Poznan, Poland
- Department of Psychiatry and Psychotherapy, Psychiatric Hospital, University of Zurich, Zurich, Switzerland
- Department of Psychotic spectrum disorders, Moscow Research Institute of Psychiatry, Moscow, Russia
- Istanbul Faculty of Medicine, Istanbul University, Psychotic Disorders Research Program, Istanbul, Turkey.
